# Genome-Wide Identification of Cotton (*Gossypium* spp.) Glycerol-3-Phosphate Dehydrogenase (GPDH) Family Members and the Role of *GhGPDH5* in Response to Drought Stress

**DOI:** 10.3390/plants11050592

**Published:** 2022-02-22

**Authors:** Jialiang Sun, Hua Cui, Bingjie Wu, Weipeng Wang, Qiuyue Yang, Yaxin Zhang, Song Yang, Yuping Zhao, Dongbei Xu, Guoxiang Liu, Tengfei Qin

**Affiliations:** 1 Key Laboratory of Tobacco Improvement and Biotechnology, Tobacco Research Institute of Chinese Academy of Agricultural Sciences, Qingdao 266100, China; sjl5845@126.com; 2 College of Agriculture, Liaocheng University, Liaocheng 252059, China; wbj8258033@163.com (B.W.); wweipeng125@gmail.com (W.W.); yqiuyue@163.com (Q.Y.); 18339511268@163.com (Y.Z.); ysyangsong111@163.com (S.Y.); zzyp000@126.com (Y.Z.); 3 Key Laboratory of Cell and Gene Circuit Design, Shenzhen Institute of Synthetic Biology, Shenzhen Institute of Advanced Technology, Chinese Academy of Sciences, Shenzhen 518055, China; hua.cui@siat.ac.cn; 4 College of Agronomy, Sichuan Agricultural University, Chengdu 611130, China

**Keywords:** drought stress, replication events, transcriptional expression profiles, protein-protein interaction network, stomatal aperture

## Abstract

Glycerol-3-phosphate dehydrogenase (GPDH) is a key enzyme in plant glycerol synthesis and metabolism, and plays an important role in plant resistance to abiotic stress. Here, we identified 6, 7, 14 and 14 GPDH genes derived from *Gossypium arboreum*, *Gossypium raimondii*, *Gossypium barbadense* and *Gossypium hirsutum*, respectively. Phylogenetic analysis assigned these genes into three classes, and most of the genes within the family were expanded by whole-genome duplication (WGD) and segmental duplications. Moreover, determination of the nonsynonymous substitution rate/synonymous substitution rate (*Ka/Ks*) ratio showed that the GPDH had an evolutionary preference for purifying selection. Transcriptome data revealed that GPDH genes were more active in the early stages of fiber development. Additionally, numerous stress-related cis-elements were identified in the potential promoter region. Then, a protein–protein-interaction (PPI) network of *GPDH5* in *G. hirsutum* was constructed. In addition, we predicted 30 underlying miRNAs in *G. hirsutum*. Functional validation results indicated that silencing *GhGPDH5* diminished drought tolerance in the upland cotton TM-1 line. In summary, this study provides a fundamental understanding of the GPDH gene family in cotton, *GhGPDH5* exerts a positive effect during drought stress and is potentially involved in stomatal closure movements.

## 1. Introduction

Agricultural crops are constrained by their natural state of entrenchment, with adverse environments substantially impacting their growth and development, and are highly susceptible to diverse abiotic stresses. Abiotic stress includes low temperatures, high temperatures, drought, salinity and flooding, which are detrimental to their growth and development, leading to injury, damage and death. These harsh growing conditions have led to significant reductions in crop yields, with drought alone affecting 45% of the world’s agricultural land [1,2]. However, the world’s population is increasing rapidly and is expected to reach 9 billion by 2050 [3]. In light of such a massive population size, areas that produce crops under severe weather must substantially increase their yields, and the crops produced must also adapt to the climate. Thus, further research on adaptive crops is necessary to maintain world agricultural production [4].

Glycerol (propane-1, 2, 3-triol) is an essential intermediate in organism metabolism and is present in all natural fats and oils in the form of fatty esters [5]. Studies have found that *Arabidopsis thaliana* mutants that cannot decompose glycerol accumulate the molecule, which significantly enhances the resistance of seedlings to abiotic stress [6,7], implying that glycerol plays an important role in the plant stress response. Previous studies have found that glycerol-3-phosphate dehydrogenase (GPDH) is a key enzyme in glycerol synthesis and metabolism in plants, acting on the donor CH-OH group with NAD^+^ or NADP^+^ as the acceptors. GPDH can hydrogenate the intermediate product of glycolysis, dihydroxyacetone phosphate, to glycerol 3 phosphate (G3P), as well as maintain the transmembrane redox potential of mitochondria [8,9], which is also a precursor for the synthesis of glycerol and triglycerides (TAGs) [10]. Five GPDH genes have been identified in *Arabidopsis*: two cytosolic NAD^+^-dependent genes [11], two plastid NAD^+^-dependent genes [12] and one mitochondrial FAD-linked gene [13]. Mitochondrial GPDH and cytoplasmic GPDH work together to maintain dynamic intracellular G3P homeostasis and participate in mitochondrial energy metabolism, while plastid GPDH is mainly involved in the synthesis of liposomes, and cytoplasmic GPDH plays a more critical role in cytoplasmic TAG synthesis [14]. A similar phenomenon was found regarding cytoplasmic salt in algae: high-salt stress induced high GPDH expressions, and their expression levels were highly inhibited under low-salt stress [15]. In maize, an important commercial crop, the GPDH transcription level was found to be highly induced in maize seedlings under abiotic stress, indicating that GPDHs play different roles in maize abiotic stress responses [16].

Cotton is an important fiber and oil crop, accounting for 35% of the total fiber used worldwide [4], which is cultivated in approximately 150 countries and provides a significant portion of income for nearly 100 million households worldwide [17,18]. The commercial cotton species under mass cultivation are *G. hirsutum* and *G. barbadense*, among them, the annual production of upland cotton accounted for more than 90% of all cotton, the sea island cotton between 3–4% [19]. *G. arboretum and G. raimondii* are diploid species with A and D genomes, respectively, while *G. hirsutum* and *G. barbadense* are allotetraploid species consisting of two sets of subgenomes: At and Dt. Furthermore, cotton shows excellent stress tolerance in the face of drought and salinity stress; it is a pioneer crop for the study of plant drought stress and salt stress [20]. Researchers have investigated the GPDH gene in *Arabidopsis*, algae [21], oilseed rape [14] and maize [16], and no corresponding reports exist for cotton; GPDH is undoubtedly a valuable research object in stress resistance studies. In this study, 6, 14, 14 and 7 GPDH family members were identified in *G. arboreum*, *G. barbadense*, *G. hirsutum* and *G. raimondii*, respectively. Bioinformatics analyses were carried out on their gene structures, conserved motifs, structural domains, phylogenies and collinearities, and the targeted miRNA and protein networks of these families were predicted. In addition, to deeply explore its role in abiotic stress, the function of *GhGPDH5* was verified, enhancing the research basis for better studying the function of GPDH.

## 2. Results

### 2.1. Identification and Fundamental Analysis of GPDH Genes from Diploid and Tetraploid Cotton

**Figure 1:**
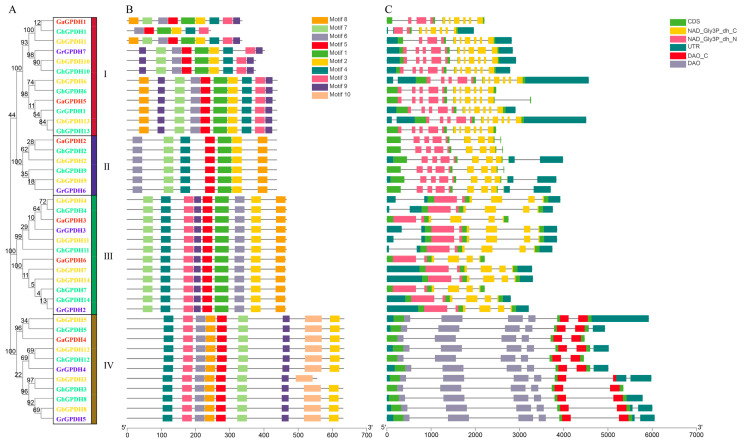
Phylogenetic relationships, motifs and structures of GPDH genes in cotton. (**A**) Evolutionary relationships. The evolutionary tree was constructed with 41 GPDH members in cotton. The four groups are divided by different colors, and different colored IDs represent different cotton species. (**B**) Ten conserved motifs were identified in cotton, with the different colored boxes representing different motifs. (**C**) Genetic structure and conserved domain information.

There are 41 GPDHs in cotton, including 6 in *G. arboreum*, 14 in *G. barbadense*, 14 in *G. hirsutum* and 7 in *G. raimondii*. For convenience, GPDH genes were renamed *GaGPDH1~GaGPDH6*, *GbGPDH1~GbGPDH14*, *GhGPDH1~GhGPDH14* and *GrGPDH1~GrGPDH7* according to their chromosomal positions. The molecular masses of the family proteins ranged from 26.61 kDa to 69.02 kDa, with an average molecular mass of 52.88 kDa. The isoelectric points ranged from 5.15 to 9.47, with an average value of 7.08. The number of amino acids ranged from 244 to 634, with an average of approximately 482 (Table 1). According to their evolutionary relationships (Figure 1A), 41 GPDH genes in cotton were classified into four groups, including 12 in group I, 6 in group II, 11 in group III and 12 in group IV. The average numbers of amino acids in groups I through IV, as well as their molecular masses, showed an increasing trend. However, the average isoelectric point in group II was the highest, while those of group IV and group I were the lowest. In addition, we performed subcellular localization analyses of GPDHs, revealing basically consistent subcellular localization in cotton and other species, but the cotton GPDH genes were mapped to the plasma membrane in two accessions.

### 2.2. Structural Characterizations and Conserved Motif Analyses of GPDH Genes

Motifs are conserved regions on genes and can be invaluable for deeply understanding gene structure. To further study the structures of GPDH gene family members, we uploaded the cotton GPDH protein sequences to the MEME website and identified the 10 most conserved motifs (Figure 1B), with each gene containing up to 9 different conserved motifs and at least 6 conserved motifs. All 10 motif sequences are provided in Supplementary Figure S2. Motifs 2/4/5/6 were present in every gene, motifs 7/8 were absent in only *GhGPDH1*, and motif 2 was absent in only *GbGPDH3*. In addition, motif 1 was not found in group IV, motif 3 was absent in group II, and motif 10 was found in only group IV. Moreover, in group I, all nine genes contained two copies of motif 9.

As shown in Figure 1C, the number of exons in the four groups ranged from 5 to 11, with the differences being minor. The exon numbers in groups II, III and IV, but group I, were consistent and were 9, 5 and 6, respectively. Moreover, cotton GPDH genes were also shown to have the typical bi-domain, which is consistent with previous reports. Specifically, groups I, II and III are NAD^+^-dependent GPDHs, containing NAD_Gly3P_dh_C(PF07479) and NAD_Gly3P_dh_N(PF01210) domains, while group IV is FAD-dependent GPDH that containing DAO_C(PF16901) and DAO(PF01266) domains. Our study confirmed that in cotton, the GPDH family only had the same conserved functional domains as *Arabidopsis*. 

### 2.3. Chromosomal Localization of GPDH Genes in Four Cotton Species

**Figure 2:**
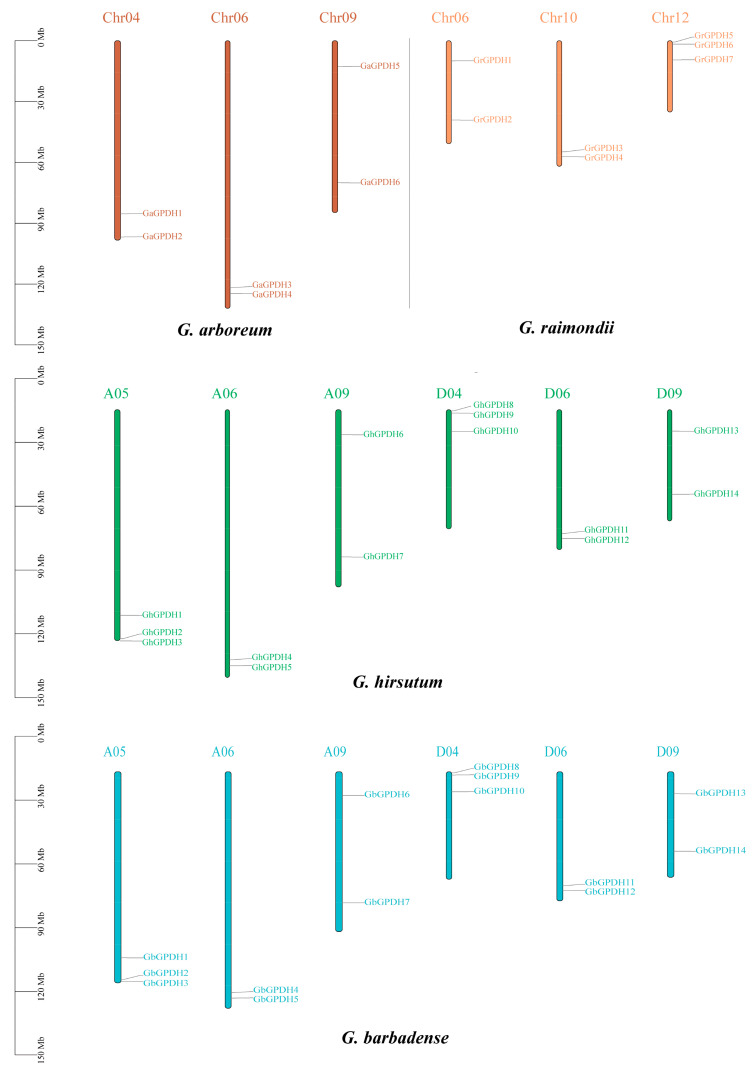
Distribution of cotton GPDH on chromosomes. The scale bar represents the length in megabases (Mb), and the different colors represent different cotton species.

Genetic evolution is complex and variable, and chromosomal deletions, duplications, inversions and translocations can greatly affect the process of gene evolution. Therefore, we constructed the GPDH family chromosome localization map (Figure 2) in this study. In diploid *G. arboreum* and *G. raimondii*, GPDHs were distributed on three chromosomes, respectively. *G. arboreum* has two family genes on each of chromosomes 04, 06 and 09, while *G. raimondii* has two family genes on chromosomes 06 and 10 and three genes on chromosome 12. In the two cultivars of cotton, GPDHs were distributed on six chromosomes, and the map showed that the distributions of GPDH family genes in the two allotetraploid cultivars were identical. At the same time, the positional permutation information of GPDH genes from subgroups A and D, remained fundamentally consistent with the two diploid ancestral species. Notably, analysis of the distribution of GPDHs at chromosomal locations revealed that chromosome A05 of *G. hirsutum* and *G. barbadense* was more similar to Chr04 of *G. arboreum*, but chromosomes D04, D06 and D09 of the D subgenome were more comparable to Chr12, Chr10 and Chr06 of *G. raimondii*. Our study suggests that the evolution of GPDH in cotton is subject to chromosomal variation.

### 2.4. Replication Events and Phylogenetic Analysis

**Figure 3:**
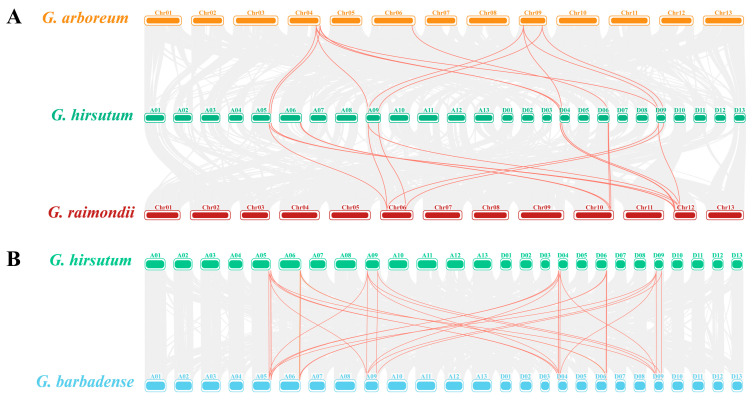
Collinearity analysis. The grey lines in the background represent the collinearity of the entire genome and the red lines represent gene pairs in the GPDH family. Chromosomes of different cotton species are represented by different colors. (**A**) The collinearity between *G. hirsutum* and two diploid cotton species. (**B**) The collinearity between *G. hirsutum* and *G. barbadense*.

Most plants have undergone evolutionary events such as genome-wide duplication and large-scale duplication [22]. The ancestors of diploid cotton *G. arboreum* and *G. raimondii* provided A and D subgenomes for the current allotetraploid cultivars of *G. barbadense* and *G. hirsutum*, respectively [23]. Our results showed that essentially twice as many GPDH genes in the two tetraploid cotton plants as in the two diploid cotton plants. Collinearity analysis of GPDH members in cotton (Figure 3) revealed that 41 GPDH members in cotton were mostly derived from whole-genome duplication or segmental duplication (Table S1). Two members (*GbGPDH4/5* and *GhGPDH3/4*) in each of G. *hirsutum* and *G. barbadense* were dispersed. The same for *GrGPDH7*, and these genes may have derived from transposons. In addition, *GbGPDH9* in *G. barbadense* is proximal, meaning that may be derived from small-scale transposition, tandem replication and the insertion of several other genes.

To better recognize *G. hirsutum* replication events, we identified all 37 homologous gene pairs in *G. hirsutum* and compared them with those in the other three cotton species. We calculated the levels of *Ka* and differences in *Ks* (Table S2). *Ka/Ks* = 1.0 indicated pseudogenes caused by neutral selection, *Ka/Ks* < 1 indicated purifying selection, and *Ka/Ks* > 1 indicated positive selection and accelerated evolution. The *Ka/Ks* ratios of all duplicate gene pairs were less than 1 (Table S2), demonstrating that GPDH gene purification selection existed during cotton evolution.

**Figure 4:**
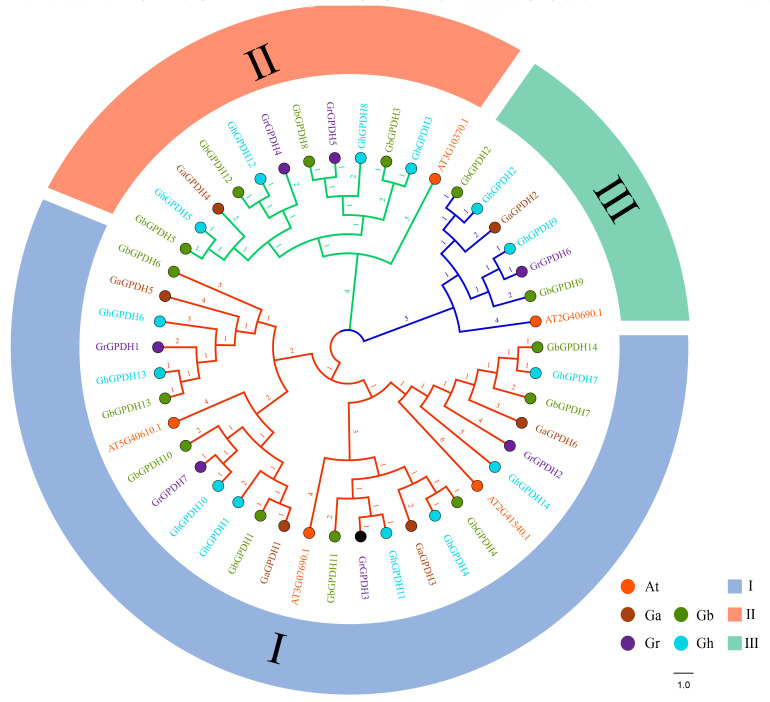
Phylogenetic tree of GPDH family members in cotton and *Arabidopsis*. At, Ga, Gb, Gh and Gr are represented by circles that are filled with orange, brown, green, blue and purple, respectively. Light purple, light orange and light green were used to distinguish the three groups. The branch label shows time which is scaled by a factor of 1.0.

Similarly, we constructed an evolutionary tree with 6 GPDH genes from *G. arboreum*, 14 GPDH genes from *G. barbadense*, 14 GPDH genes from *G. hirsutum*, 7 GPDH genes from *G. raimondii* and 5 GPDH genes from *A. thaliana* (Figure 4). According to the results, 46 GPDH genes in cotton and *Arabidopsis* were divided into three groups. Genes in the same group were more closely related, and group I was the largest subgroup, containing 27 members, while groups B and C included 12 and 7 members, respectively. The three diploid species, including *Arabidopsis*, were grouped with only one gene, while the two tetraploid cotton species each had two genes. At the same time, at least 1 gene was classified in each group of the four cotton species, indicating no significant deviations during the rapid evolution of cotton. These findings suggested that the evolutionary development of the GPDH family in cotton was highly consistent with that in *Arabidopsis*.

### 2.5. Gene Expression Profile Analysis of GPDH Family Members

**Figure 5:**
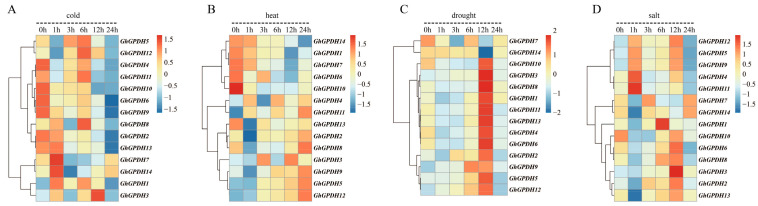
Transcription expression profiles of GPDH family genes in *G. hirsutum* under different stress treatment conditions. (**A**–**D**) are the transcriptional profiles of GPDH family genes under cold, heat, drought and salt stresses, respectively. Genes that were not expressed at all times were excluded.

It is widely known that gene expression is spatially and temporally specific, and it is thus necessary to analyze the regulation of gene expression from a transcriptomic perspective. Previous reports have shown that GPDH plays an important role in abiotic stress, we constructed a gene expression map of GPDH family genes in upland cotton. The heatmap (Figure 5) shows that the expression profile of GPDH family genes was consistent in response to drought stress compared with that under other stress treatments. Except for *GhGPDH1* and *GhGPDH14*, all of the genes showed a trend of first increasing and then decreasing and peaked at 12 h post treatment. Under cold stress, GPDHs exhibited higher expression in the first 6 h and then showed decreased expression. In addition, the expression trends of *GhGPDH5/9/12* under heat, drought and salt stress conditions were basically the same; that is, the expression level gradually increased as the treatment time was extended and then decreased after peaking. These findings showed that the levels of GPDHs were significantly altered as the stress duration increased and had similar patterns under osmotic stress.

**Figure 6:**
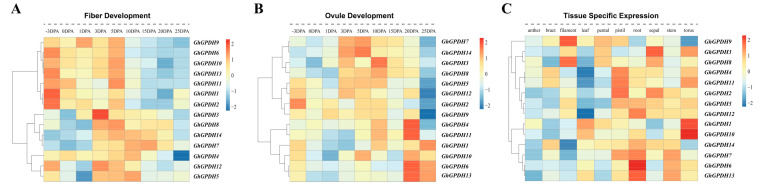
Transcriptional expression profiles of GPDHs in ovules, fibers and tissues of *G. hirsutum* at different developmental stages.

Additionally, to elucidate the expression of GPDH genes in tissues and their effects on fiber development, we also constructed heatmaps of different tissues and different developmental periods in upland cotton. The heatmap (Figure 6C) showed that the expression trends of GPDHs were low in anthers, bracts and leaves. *GhGPDH9/8* in filaments, *GhGPDH2/4/11* in the pistil, and *GhGPDH2/3* in the calyx were highly expressed. In roots, *GPDH6* expression tended to be extremely high, and the *GhGPDH12/13* expression levels were also high, in the torus, *GhGPDH1/10* expression was consistently high. During fiber development (Figure 6A), *GhGPDH1/2/6/9/10/11/13* were highly expressed from −3 DPA to 5 DPA, while *GhGPDH3/4/5/7/8/12/14* were highly expressed from 3 DPA to 20 DPA. In addition, the two stages of 3DPA and 5DPA corresponded to the initiation and extension stages, respectively, of cotton fiber development, during which almost all the genes in the family were highly expressed, indicating that the GPDH family is quite active during the initial stage of fiber development. In ovules (Figure 6B), the expression levels of *GhGPDH2/3/4/5/7/8/9/12/14* were higher from 3 DPA to 20 DPA, and after 20 DPA, *GhGPDH1/4/6/10/11/13* were expressed at higher levels. This evidence suggests that GPDHs may also play an essential part in cotton fiber development.

### 2.6. Cis-Element Analysis of GPDH Promoter Region

**Figure 7:**
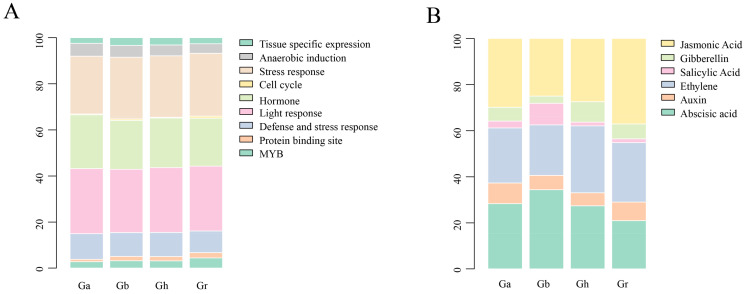
Distribution of cis-elements of GPDH members in *G. hirsutum*. The block size represents the proportion, and different rectangles are filled with different colors. (**A**) Inducible cis-elements. (**B**) Hormone-related cis-elements.

The 2000 bp sequence upstream of the coding sequence (CDS) is considered the promoter region, which can activate RNA polymerase and accurately bind to template DNA to initiate transcription with specificity. Inducible promoters can respond immediately to stimulus signals and regulate the expression of related genes under specific environmental and stimulus conditions. Total nine types of cis-elements were identified in cotton, and their number distribution in different species were basically consistent (Figure 7A); 6 are related to injury defense, 4 are related to MYB transcription factors, 7 are related to light response, 5 are related to stress response, and 3 are related to metabolic regulation. In addition, there were 12 hormone-related elements (Figure 7B), namely, the TGA element, ERE, ABRE4, TCA element, P-box, CGTCA motif, AuxRR core, ABRE3a, AT~ABRE, TATC box, GARE motif and TGACG motif. These findings confirmed that the promoter region of GPDHs is enriched with numerous hormonal response elements and stress response elements, which may directly or indirectly impact the expression of genes critical for cotton growth and development and thus, how cotton performs in response to biotic and abiotic stresses.

### 2.7. Protein Interaction Network Analysis of GPDH5 in G. hirsutum

**Figure 8:**
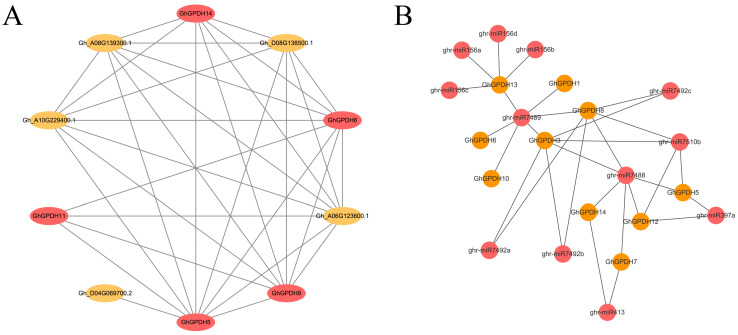
Network diagram of *GPDH5* protein interactions and miRNAs targeting the GPDH family in *G. hirsutum*. (**A**) Interactive relationships of *GPDH5* in *G. hirsutum*. (**B**) Network of miRNAs associated with GPDH genes in *G. hirsutum*.

Because there are only a few examples of a single protein playing a role in plant responses to various stresses, family gene interactions are extremely important. However, no database available for cotton can directly predict protein interactions of a particular gene. In this study, the homologous gene *GhGPDH5* of *AT3G10370 (SDP6)* was used to predict the protein interactions in *Arabidopsis* (Figure S1), and these interaction protein sequences were replaced with homologous gene sequences from *G. hirsutum* to predict the *GhGPDH5* interaction proteins (Figure 8A). In *Arabidopsis*, 10 pairs of SDP6 protein interactions were identified, and the average confidence level reached the high percentage of 96.62%; the lowest value was 94.9%, and the highest value was 99.6% (Table S3). Moreover, all the AtGPDHs were involved in the predicted interactions, thus indicating the high reliability of the results. In *G. hirsutum*, a total of 9 genes interacting with *GhGPDH5* and 33 interactive relationships were identified, and 4 of the 9 genes were also members of the GPDH family. All these interactions have profound implications for future protein function studies. 

### 2.8. Potential miRNAs That Target to GhGPDHs

With the continuous development of sequencing technology, omics research has made great progress, and an increasing number of plant miRNAs have been found to be involved in various developmental processes. Therefore, studying miRNAs targeting GPDH family members is helpful for understanding the role of the GPDH family in abiotic stress responses. In this study, a total of 30 interactions were predicted (Figure 8B), and involved 12 miRNAs and 10 family genes. Among them, multiple miRNAs targeted more than one family gene. Both *ghr-miR7488* and *ghr-miR7489* targeted 6 GPDH family genes, and *GPDH3/8/13* were associated with 6, 6 and 5 different miRNAs, respectively. Two highly repetitive sequence combinations, *miR156* and *miR7492*, were also predicted. MiR156 is a small RNAs that has been intensively researched, and several reports have confirmed that it has an active role in abiotic stresses [24,25]. Our results implied that GPDHs may interact with miR156, which most likely explains the excellent performance of GPDH in response to abiotic stresses.

### 2.9. Preliminary Validation of GPDH5 Function in G. hirsutum

**Figure 9:**
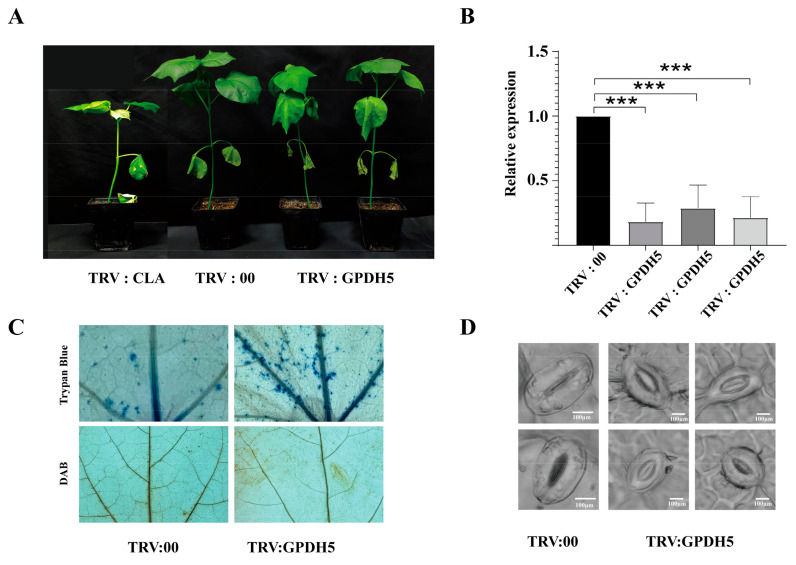
Functional verification of *GPDH5* under drought stress. (**A**) Whole plant phenotypes of gene silenced plants. A plant with *CLA*-silencing, negative control plant and two plants with *GhGPDH5*-silencing are shown from left to right. (**B**) Relative expression levels in silenced and TRV:00 plants. Data analysis was performed using one-way ANOVA by GraphPad Prisim 8, *** represents the extremely significant difference between negative control and experimental plants. The error bars show the standard deviation of three technical replicates (**C**) TRV:00 and TRV:*GPDH5* after DAB and trypan blue staining. (**D**) Comparison of stomatal apertures between TRV:00 and silenced plants. Microscope magnification is 640x.

Various genes showed a clear regulatory response to cotton being exposed to drought stress according to the heatmap, suggesting a potential role for GPDH genes in response to drought stress. However, these genes were unfortunately expressed at low levels, and we thus selected *GhGPDH5*, which was expressed at a higher level, to further verify the role of GPDHs in response to drought stress. We carried out a virus induced gene silence (VIGS) assay to verify the role of *GhGPDH5* in TM-1 (primer information shown in Table S4). Compared with the control, the plants with *GhGPDH5* silencing displayed obvious phenotypic differences after a period of drought treatment. The tolerance of the silenced cotton seedlings to drought stress was significantly weakened (Figure 9A), and the leaves of the silenced plants were more wilted than those of plants treated with the empty vector or the wild sequence. The experimental results were consistent with our expectations. Subsequently, we examined cell necrosis on the leaves by trypan blue staining, revealing that cell death was induced in TRV:*GPDH5* leaves and slightly induced in the TRV:00 (pYL192:pYL156:00) leaves (Figure 9C). Because the peroxides in cells are closely related to GPDH [26,27], we detected the catalase content in leaf cells by 3,3-diaminobenzidine (DAB) staining, demonstrating that the number of lesions in silenced plant leaves was increased (Figure 9C). Stomata are important channels and key factors for transpiration in plants [28,29], and the decrease of water content in plants after drought stress most likely affect the occurrence and movement of stomata. Therefore, we observed the stomata of control and silenced plants under the microscope, and the results are shown in Figure 9D. The stomata of the control plants were closed tightly, while those of the silenced plants were open to a certain extent. To further validate these results, we performed qPCR. And the expression level of *GhGPDH5* was always significantly lower in the silenced plants than in control samples (Figure 9B). These results indicated that *GhGPDH5* played a positive role in the drought stress response of *G. hirsutum*.

## 3. Discussion

Generic improvement of cotton is time consuming and difficult. With the innovation of new sequencing technologies, genomic data for *G. arboreum*, *G. barbadense*, *G. hirsutum* and *G. raimondii* have been released, assembly quality has been improved, and more complete genome data are now available. These advancements, combined with the vigorous development of bioinformatics, abundant available genome information and analysis software, have greatly assisted us with our research and analysis. Among the long genetic improvement history of cotton, drought and salt are two of the most inescapable abiotic stress categories that seriously affect cotton yield and fiber quality. In the first stage of drought and salinity stress during cotton growth, osmotic stress is the main manifestation [30]. When plants are stressed by osmotic, the compatible solute concentrations in the cell increase to maintain the intracellular osmotic pressure balance [7]. It has been reported that glycerol can be specifically retained in the cytoplasmic solute under abiotic stress [31]. In a series of experiments by Zhao et al., zmGPDH1 confers salinity and osmotic tolerance in *Arabidopsis* by regulating glycerol synthesis, stomatal closure, cellular redox and ROS homeostasis, and overexpression of GmGPDH12 helped maintain a constant respiratory rate under salt or osmotic stress [32,33]. In view of the research progress on GPDH members involved in plant breeding and abiotic stress responses, the identification of GPDH members in cotton has important implications for the selection of drought and salt tolerant cotton plants.

Transcription factors (TFs) are pivotal modulators of crop improvement, and facilitate the activation of regulatory mechanisms in plants [22,34]. The promoter region of cotton GPDH genes, contains numerous transcription factor binding sites, including multiple MYB-associated transcription factor recognition sites, and MYB has been shown to be associated with the abiotic stress response in plants in several studies [35,36,37,38]. Likewise, the hormone constitutes one of the important factors in affecting cotton growth and development, and abundant stress-related hormone response cis-elements further illustrate the potential of GPDH in coping with abiotic stresses. In contrast to transcription factors, motifs are more practical representations of consensus elements in biological sequences, permitting a more detailed description of the variability of each locus. Various transcription factors harbor conventional motif genres responsible for binding to DNA and individual TFs typically recognize collections of similar DNA sequences [39]. In our result, motifs 2/4/5/6 were identified in each cotton GPDH gene, so we speculated that these four motifs are the intrinsically conserved motifs of GPDH, and no motif 1 was found in group IV but the relative replacement was conserved motif 10; it precisely corresponded to the double structural domain feature of GPDH gene. In consideration of the dual structural domain features of the GPDH family [16,21], according to the phylogenetic division, group IV corresponds to FAD-dependent GPDH and group I/II/III corresponds to NAD-dependent GPDH. We conjectured that motif10 may be an important motif on the domains of DAO_C (PF16901) and DAO (PF01266), and motif1 may be a key motif on the domains of NAD_Gly3P_dh_C (PF07479) and NAD_Gly3P_dh_N (PF01210). 

Polyploidy is the main mechanism of plant formation and adaptation to the external environment [40], and gene duplication leads to the functional differentiation and diversification of genes, which is thought to be the major driving force of evolution [41]. As an allotetraploid, *G. hirsutum* has experienced multiple gene replication events, and the number of genes have increased continuously throughout its evolution, making it one of the ideal species for studying polyploid formation in the whole-genome evolution process [42]. We identified 41 GPDH genes in cotton, with almost twice as many family members being observed in the two tetraploid cotton plants as in their two diploid ancestors. Meanwhile, in each group, GPDHs were twice as numerous in tetraploid cotton as in diploid. There were no tandem duplications of GPDH family members in cotton, most of the family genes were derived from whole-genome replication and fragment duplication, and only a few individual genes originated from transposon translocation effects. These observations suggest that whole-genome duplication and fragment duplication dominated the rapid evolution of the GPDH family in cotton. In the phylogenetic tree constructed with *Arabidopsis*, the 41 GPDH members were divided into three groups, which was consistent with the division of three different GPDH genes in *Arabidopsis*. We calculated the ratio of *Ka* to *Ks* using homologous gene pairs in cotton, and all ratios were less than 1, indicating that the evolution of GPDH family members tends to be geared towards purifying selection, which further explains the uniform chromosomal distribution of GPDHs in the two tetraploid cotton lines. In *Dunaliella salina*, some GPDH genes contain not only the canonical NAD^+^-GPD domain but also a unique domain, the haloacid dehalogenase (HAD)-like superfamily domain (PF12710), in their N-terminal region [21]. In cotton, all GPDH family genes were found to contain only the classical NAD_Gly3P_dh_N, NAD_Gly3P_dh_C or DAO, DAO_C functional domains, which correspond to NAD^+^-dependent and FAD^+^-dependent phenotypes of *Arabidopsis*, respectively. 

Analyses of the expression profiles of GPDH members in *G. hirsutum* under four abiotic stress conditions showed that most GPDH members were expressed at relatively consistent levels under drought and salt stresses associated with osmotic stress, as their levels first increased and then decreased after stress. This trend may have been attributed to the stress response of plants after receiving the stimulus signal. At that time, GPDH expression was active, and the osmotic pressure in the cytoplasm was reduced via the mass production of glycerol, thus completing the stress response. *GmGPDH12* also showed a significant transcriptional response to NaCl and mannitol treatments [33]. Similar results were also shown in *Brassica napus* in response to stress [43]. In addition, our results showed that the GPDH family was more active during the early development of cotton fiber, which may have been due to the need for considerable energy during this period. Therefore, GPDH may also indirectly affect cotton fiber quality by affecting energy metabolism.

Functional validation results were consistent with experimental design predictions, with TRV:*GhGPDH5* plants showing a significant reduction in their ability to cope with drought compared to negative control plants. This is not the only time this result has been reported. Ying et al. have taken the overexpressed and silenced *GmGPDH12* in soybean hairy roots, which confirmed that the GPDH increased the plants tolerance and sensitivity to salt and osmotic stress, respectively [33]. In *Saccharomyces cerevisiae*, *GPD2* is highly expressed under hypoxic conditions to synthesize glycerol and consume NADH in cells and thereby maintain the REDOX balance [44,45]. As oxidoreductase, GPDH competes with catalase. To maintain their own REDOX balance, plant cells with GPDH silencing will accelerate the production of catalase to consume NADH in the cells, which explained the increase in catalase contents in the leaves of silenced plants. GPDH is an important player in maintaining the NAD^+^ homeostatic environment in plants, our research echoes previous studies.

Stomata influence the rate of water evaporation from plants, and the amount of water loss affects the plant’s tolerance to drought stress. Except for some bryophytes with highly developed microtubular tissues [46], the degree of plant stomatal opening size is closely related to water loss and also reduces pathogens entering through the pores, this is a key feature for water conservation and disease prevention [47,48,49]. Our results confirmed that after being subjected to drought stress, the stomata of TRV:*GPDH5* plants had significantly larger stomatal openings than those of the negative control, such a phenomenon is likely to cause accelerated water loss in the silenced plants. *ZmGPDH1* reportedly contributes to *Arabidopsis* salinity and osmotic tolerance by modulating glycerol production, stomatal closure, cellular redox and ROS homeostasis [32]. It may be linked to ABA, which plays a key role in regulating stomatal movement [50,51,52]. Recent studies have shown that the exogenous application of NAD leads to an increase in the ABA content [53]. At the same time, an increase or decrease in NAD leads to a decrease in plant stomatal number and density, suggesting that NAD may indirectly affect stomatal development by influencing ABA synthesis and signal transduction [54]. 

Above all, our results highlight the potential role of the GPDH family in coping with drought stress as well as in stomatal opening and closing movements. It is the first genome-wide identification of GPDH genes in cotton, and our study is of great relevance for researchers aiming to better understanding of GPDH functions.

## 4. Materials and Methods

### 4.1. Identification of GPDH Genes in Cotton

Four reliable genome assemblies (*G. arboreum*, CRI; *G. raimondii*, JGI; *G. barbadense*, NAU; *G. hirsutum*, CRI) were obtained from the cotton functional genome database (CottonFGD, https://cottonfgd.org/, accessed on 1 February 2022) [55]. The sequences of 5 AtGPDHs were downloaded from The *Arabidopsis* Information Resource (TAIR, https://www.arabidopsis.org/, accessed on 20 January 2022) [56]. Initial sequences were searched in the local library through BLAST using *A. thaliana* homologous genes as queries. Then, hmmerscan of HMM (version 3.3) [57] was used to verify the final sequences. The molecular masses and isoelectric points of cotton GPDH proteins were obtained from ExPASy (http://web.expasy.org/, accessed on 20 January 2022) [58,59]. The CELLO program (http://cello.life.nctu.edu.tw/, accessed on 20 January 2022) was used to predict subcellular localization [60,61].

### 4.2. Gene Structures and Conserved Motifs Prediction

The location information regarding cotton GPDH genes was obtained from the GFF file information corresponding to their respective genomes. Conserved motif structure information was obtained from MEME [62], structural domain information was confirmed in NCBI-CDD, and the Pfam database was also used. TBtools software was used to display the final graphics [63].

### 4.3. Phylogenetic Analysis

Multiple sequence alignment of GPDH protein sequences from cotton and *A. thaliana* was performed in MUSCLE [64] with default parameters. A phylogenetic tree was constructed using MEGAX [65] software with the maximum likelihood (ML) [66] method, and the reliability was assessed with 1000 bootstrap replications. Graphs were generated and beautified in FigTree v1.4.4 and AdobeIllustratorCS6.

### 4.4. Chromosomal Distribution and Collinearity Analysis

Information on each chromosome in cotton was obtained from the gff files corresponding to the respective genomes. Variable splices of the four genome protein sequences were removed, and BLASTP databases of the four cotton species were constructed locally. The four cotton species were then compared, and collinearity analysis was performed using MCScan [67] to collate the collinearity relationship among *G. arboreum*, *G. raimondii* and *G. hirsutum* as well as the two allotetraploid cultivated cotton lines.

### 4.5. Gene Expression Analysis of the GPDH Family in G. hirsutum

Transcriptome data were downloaded from the cottonFGD website, and heatmaps were plotted on the Rstudio [68] platform using the R package ‘pheatmap’. In addition, family genes with expression profiles of 0 at all stage points were removed to improve the graph presentation, and all gene expression levels were normalized (taking log2 (fpkm+1)).

### 4.6. Analysis of Cis-Elements in the Promoter Region

A 2000 bp sequence upstream of the gene was extracted, and the possible cis-acting elements were predicted in PlantCARE (http://bioinformatics.psb.ugent.be/webtools/plantcare/html/, accessed on 20 January 2022) [69] and visualized in Rstudio.

### 4.7. MiRNA Prediction of GPDH Genes in G. hirsutum

The *G. hirsutum* miRNA database was downloaded from the plant microRNA database [70] and uploaded to the psRNATarget (2017 release) website (https://www.zhaolab.org/psRNATarget/, accessed on 1 February 2022) [71] to obtain potential miRNAs. Cytoscape 3.7.2 was used for visualization.

### 4.8. Construction of the GhGPDH5 Protein-Protein Interaction Network

The sequences of homologous GPDH genes in *Arabidopsis* were uploaded to the STRING (https://string-db.org/, accessed on 1 February 2022) [72] website to obtain the interaction network information regarding GPDH genes in *Arabidopsis*, and then all the interaction protein sequences obtained in *Arabidopsis* were then aligned to the *G. hirsutum*. genome. After sorting, the *Arabidopsis* interaction protein IDs were replaced with *G. hirsutum* IDs to predict the interaction proteins in *G. hirsutum* [73], which were visualized using Cytoscape 3.7.2.

### 4.9. Gene Function Validation and Quantitative Real-Time PCR (qPCR) 

To further validate the function of GPDHs in response to drought stress, we performed a VIGS experiment [74,75,76]. All plasmid vectors used (TRV: CLA1, pYL192 (TRV-RNA1), pYL156 (TRV-RNA2)) are stored in our laboratory refrigerator. We avoided the conserved region and amplified a *GhGPDH5* fragment of 403bp in length and inserted it into the pYL156 vector to construct a recombinant plasmid TRV:*GhGPDH5*. TRV:CLA1 (cloroplastos alterados 1) was used as a positive control as previously described [77]. All the constructs were verified by enzymatic identification and DNA sequencing (Sangon, Shanghai, China). These vectors were transferred into Agrobacterium tumefaciens strain GV3101 by a heat shock method, respectively. We then placed these carriers in the LB medium supplemented with kanamycin and rifampicin (50 mg/L), and the cells were incubated in osmotic medium containing 10 mM MgCl_2_, 10 mM MES and 20 μM acetosyringone. After treatment for 3-6 h in the dark, *Agrobacterium tumefaciens* medium containing pYL156:00, TRV:*CLA1* and TRV:*GhGPDH5* was mixed with pYL192 at 1:1. 

The standard variety TM-1 (*G. hirsutum*) was used as the experimental plant, seedlings grew under the same environmental conditions (24° ambient temperature with 16 h of light and 8 h of darkness). Each plant was grown in a substrate of 1:3 mixture of vermiculite and nutrient soil and watered with a 0.5 g/L concentration of chlorinated fertilizer water. Threat treatments were applied by natural drought, and cotton seedlings were injected when they had just developed their first true leaves and were not watered for 5 days prior to injection. A 24-h dark treatment was performed after the injection, and then no more watering was done after one watering. Each recombinant vector was injected with 20 cotton seedlings in each batch of experiments, and a total of 3 batches of experiments were conducted. Forty true leaves of each TRV:00(pYL192:pYL156:00) and TRV:*GhGPDH5* plant were collected (15 days after injection). Then, 15 leaves were stained with trypan blue staining solution (solution consisting of 15 mg trypan blue, 10 mL of 85% lactic acid, 10 mL glycerol, 10 mL phenol,10 mL ddH_2_O) [78,79,80] and 15 leaves were stained with 1 mg/mL diaminobenzidine (DAB) (PH = 3.8), both for a duration of 12h [81,82]. Then, 98% alcohol was used to wash away the staining solution, and the decolorized leaves were then placed in water for photographing. In addition, five temporary slides were made using the lower epidermal cells of different leaves of TRV:00 and TRV:*GhGPDH5* each, and stomatal was observed under a microscope (SAIKEDIGITAL, Shenzhen, China).

Three sets of samples from different plants (15 days after injection) were collected as three biological replicates. The leaves of TRV:00 and TRV:*GhGPDH5* cotton seedlings were ground to powder for total RNA isolation using an EASYspin Plus Plant RNA Kit (Aidlab, Beijing, China) according to the manufacturer’s instructions. The RNA reverse-transcribed was using Trans Script One-Step gDNA Removal and cDNA Synthesis SuperMix (Trans Gen, Beijing, China). Cotton Histon3 (GhHiston3) was used as an endogenous standard control. Primers were designed using Primer Premier 6 and BioXM2.7.The RT-PCR protocol was as follows: (1) 95 °C for 5 min; (2) 95 °C for 30 s, 58 °C for 30 s, and 72 °C for 45 s for 40 cycles; and (3) 72 °C for 10 min. qPCR assays were performed on a QuantStudio 6 Flex thermocycler (Applied Biosystems, Foster City, CA, USA) using Trans Start Top Green qPCR SuperMix (Trans Gen, Beijing, China) with a total volume of 10 μL. Melt curve analysis was performed after the qPCR cycle.

## Figures and Tables

**Table 1:** Basic information of GPDH family genes in cotton.

Type	ID	Name	MW	PI	Subcellular Location	Amino Acid Numbers	Region
I	Ga04G1449	GaGPDH1	36,512.62	5.47	Plasma Membrane	336	Chr04: 85365308–85367513 (+)
	Gh_A05G359000	GhGPDH1	26,610.66	5.15	Cytoplasmic	244	A05: 96649619–96651585 (+)
	Gbar_A05G035870	GbGPDH1	36,572.71	5.46	Plasma Membrane	336	A05: 90403265–90406088 (+)
	Gorai.012G066600	GrGPDH7	44,225.84	5.79	Cytoplasmic	402	Chr12: 9501046–9503893 (-)
	Gbar_D04G006270	GbGPDH10	41,212.08	5.26	Cytoplasmic	376	D04: 9780994–9783914 (-)
	Gh_D04G066600	GhGPDH10	41,154.04	5.35	Cytoplasmic	376	D04: 10236191–10238979 (-)
	Gbar_A09G003620	GbGPDH6	48,156.2	5.7	Chloroplast	439	A09: 11430821–11435386 (-)
	Gh_A09G038400	GhGPDH6	48,126.17	5.7	Chloroplast	439	A09: 11794439–11796911 (-)
	Ga09G0401	GaGPDH5	47,514.61	6.24	Chloroplast	434	Chr09: 12758934–12762193 (-)
	Gorai.006G036200	GrGPDH1	48,098.07	5.44	Chloroplast	438	Chr06: 9950055–9952964 (-)
	Gbar_D09G003380	GbGPDH13	48,128.19	5.6	Chloroplast	438	D09: 10699107–10703615 (-)
	Gh_D09G035900	GhGPDH13	48,186.23	5.51	Chloroplast	438	D09: 10082117–10084586 (-)
II	Ga04G1991	GaGPDH2	47,231.15	9.47	Mitochondrial	437	Chr04: 96767830–96770413 (-)
	Gh_A05G409500	GhGPDH2	47,186	9.47	Mitochondrial	437	A05: 107687182–107689805 (-)
	Gbar_A05G040550	GbGPDH2	47,233.12	9.47	Mitochondrial	437	A05: 100948496–100952479 (-)
	Gh_D04G012900	GhGPDH9	47,220.1	9.32	Chloroplast	437	D04: 1567590-1570243 (+)
	Gbar_D04G001360	GbGPDH9	47,161.08	9.25	Chloroplast	437	D04: 1580690-1584522 (+)
	Gorai.012G015200	GrGPDH6	47,186.08	9.32	Chloroplast	437	Chr12: 1696170-1699874 (+)
III	Gbar_A06G017080	GbGPDH4	52,174.53	6.5	Cytoplasmic	466	A06: 107255401-107259323 (-)
	Gh_A06G186000	GhGPDH4	52,174.53	6.5	Cytoplasmic	466	A06: 117541104-117544857 (-)
	Ga06G1950	GaGPDH3	52,075.37	6.31	Cytoplasmic	466	Chr06: 121758944-121761694 (+)
	Gorai.010G191500	GrGPDH3	52,161.46	6.36	Cytoplasmic	466	Chr10: 54807050-54810899 (-)
	Gbar_D06G017760	GbGPDH11	52,161.46	6.36	Cytoplasmic	466	D06: 55366601-55370448 (-)
	Gh_D06G188200	GhGPDH11	52,147.43	6.36	Cytoplasmic	466	D06: 58158384-58162126 (-)
	Ga09G1387	GaGPDH6	51,608.48	6.4	Cytoplasmic	464	Chr09: 70045246-70047459 (+)
	Gbar_A09G013050	GbGPDH7	51,622.51	6.4	Mitochondrial	464	A09: 63713685-63716969 (+)
	Gbar_D09G012780	GbGPDH14	51,564.44	6.4	Cytoplasmic	464	D09: 38612288-38615590 (+)
	Gh_A09G139800	GhGPDH7	51,622.51	6.4	Mitochondrial	464	A09: 69189679-69191891 (+)
	Gh_D09G131000	GhGPDH14	51,564.44	6.4	Cytoplasmic	464	D09: 39773884-39776685 (+)
IV	Gorai.006G135700	GrGPDH2	51,564.44	6.4	Cytoplasmic	464	Chr06: 39159929-39163138 (+)
	Gbar_A06G018390	GbGPDH5	69,021.82	8.71	Mitochondrial	634	A06: 109949842-109955764 (+)
	Gh_A06G199100	GhGPDH5	69,022.76	8.54	Chloroplast	634	A06: 120372686-120377617 (+)
	Ga06G2073	GaGPDH4	69,003.71	8.43	Mitochondrial	634	Chr06: 124625099-124629571 (-)
	Gbar_D06G019050	GbGPDH12	68,997.76	8.12	Chloroplast	634	D06: 57729486-57734502 (+)
	Gh_D06G201500	GhGPDH12	68,999.69	7.89	Chloroplast	634	D06: 60515219-60519674 (+)
	Gorai.010G205900	GrGPDH4	68,929.65	8.3	Chloroplast	633	Chr10: 57195504-57200512 (+)
	Gbar_A05G041420	GbGPDH3	59,564.11	7.55	Chloroplast	555	A05: 101934946-101940926 (-)
	Gh_A05G417800	GhGPDH3	68,627.3	8.14	Mitochondrial	631	A05: 108672565-108677912 (-)
	Gh_D04G004200	GhGPDH8	68,605.27	8.12	Mitochondrial	631	D04: 562838-568622 (+)
	Gbar_D04G000480	GbGPDH8	68,669.4	8.43	Mitochondrial	631	D04: 553663-559666 (+)
	Gorai.012G005900	GrGPDH5	68,618.3	8.29	Mitochondrial	631	Chr12: 696866-702921 (+)

## Data Availability

The genome sequences of plant species and RNA-seq data used in our manuscript were downloaded from the CottonFGD website (https://cottonfgd.org/, accessed on 20 January 2022).

## Supplementary Materials

The following supporting information can be downloaded at: https://www.mdpi.com/article/10.3390/plants11050592/s1, Figure S1: Protein–protein interaction network prediction map of SDP6 gene in Arabidopsis; Figure S2: Sequences of the 10 motifs; Table S1: Genetic amplification phenotype of cotton GPDH; Table S2: Ka/Ks ratio of GPDH homologous genes between *G. hirsutum* and other cotton species; Table S3: Predicted results of Arabidopsis SDP6 interactions; Table S4: Experimental primer sequence.

## Abbreviations

TAIR	The Arabidopsis Information Resource
MUSCLE	Multiple Sequence Comparison by Log-Expectation
ML	Maximum Likelihood
BLAST	Basic Local Alignment Search Tool
Ka	The number of nonsynonymous substitutions per nonsynonymous site
Ks	The number of synonymous substitutions per synonymous site
DPA	Days Post Anthesis
CDS	Coding Sequence
Gh	*G. hirsutum*
Ga	*G. arboreum*
Gr	*G. raimondii*
Gb	*G. barbadense*
At	Arabidopsis
ABA	Abscisic acid
DAB	3,3′-diaminobenzidine
TPB	Typan Bue
VIGS	Virus-Induced Gene Silencing
PI	Isoelectric point
MW	Molecular Weight
TRV	Tobacco Rattle Virus
