# Supplementary figures and images for: Genome-Wide Identification of Cotton (Gossypium spp.) Glycerol-3-Phosphate Dehydrogenase (GPDH) Family Members and the Role of GhGPDH5 in Response to Drought Stress
Source: Plants (Basel). 2022 Feb 22;11(5):592. doi: 10.3390/plants11050592 (PMC8912411; doi:10.3390/plants11050592)

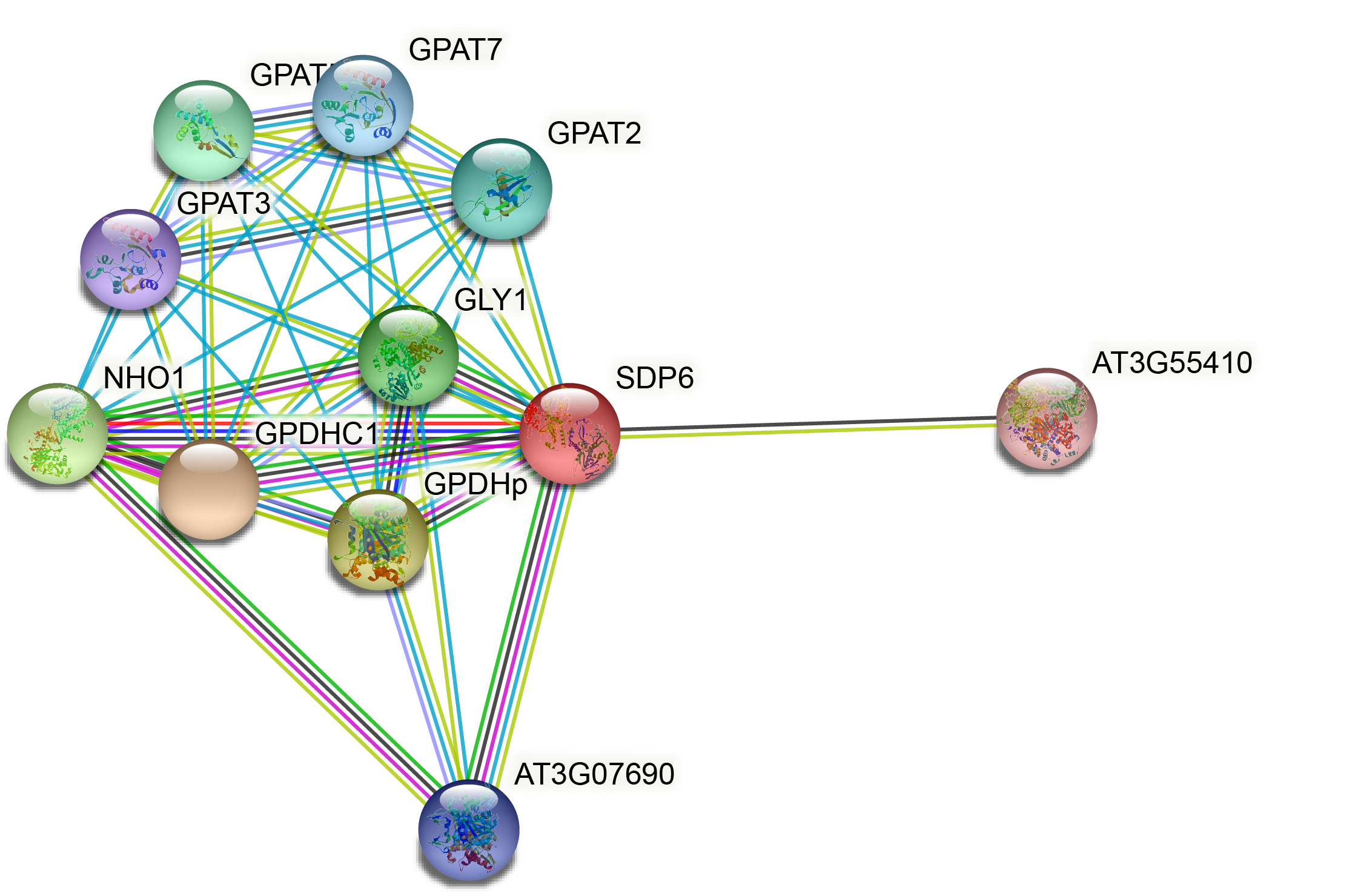

Supplement: Supplementary file 1 [file plants-11-00592-s001.zip › Supplementary File/Figure S1.png]
